# Using Social Network Sites to Boost Savoring: Positive Effects on Positive Emotions

**DOI:** 10.3390/ijerph17176407

**Published:** 2020-09-02

**Authors:** Sen-Chi Yu, Kennon M. Sheldon, Wen-Ping Lan, Jia-Huei Chen

**Affiliations:** 1 Department of Counseling and Applied Psychology, National Taichung University of Education, West District, Taichung City 403, Taiwan; 2 Department of Psychological Sciences, University of Missouri, Columbia, MO 65211, USA; SheldonK@missouri.edu (K.M.S.); bubblewind@gmail.com (W.-P.L.); tasmtw@gmail.com (J.-H.C.)

**Keywords:** savoring, positive interventions, positive psychology, social network sites, Facebook

## Abstract

Research has demonstrated that positive interventions (PIs) can be effective in enhancing well-being. Our study used Facebook to conduct a PI based on savoring. Sixty-one university students in Taiwan were randomly assigned to undergo a three-week savoring PI, and 61 participants were assigned to a no-treatment control group. The results showed significantly enhanced positive affect in the treatment group compared to the control group, in both a post-test and a final follow-up, but no significant differences between the two groups in negative affect. The treatment group also displayed significantly lower depression in the post-test, which was not maintained at the follow-up. These results indicate that, for university students, a savoring intervention via Facebook can be an effective way of enhancing positive emotions.

## 1. Introduction

Early research on psychology mostly focused on negative mental states (such as depression and anxiety) and dwelled less on the causes and consequences of positive mental states [1]. Seligman’s introduction [1] of the concept of positive psychology (PP) has led psychological researchers to attach greater importance to understanding and enhancing well-being and positive emotion [2]. Positive psychology aims to understand the positive side of human functioning, expanding research on positive behaviors, cognitions, emotions, and character traits [3]. Today, PP is an umbrella term that covers many concepts and theories regarding how humans can live better. The general objective is to help people to improve their way of being in the world, so as to be happier and more productive.

Savoring is the capacity to attend to, appreciate, and enhance the positive experiences in your life [4]. Savoring refers to the ability to take pleasure in anticipation of future positive events, to enjoy positive experiences in the present moment, and to remember past experiences with pleasure [5]. In other words, savoring involves a capacity to regulate and enhance one’s own positive emotions regarding future, present, and past positive experiences [4]. Studies have shown that a positive correlation exists between savoring and positive emotion and that savoring mediates the impact of daily positive events upon happiness [2,4,6]. Furthermore, research has demonstrated that savoring is associated with life satisfaction [4].

Positive interventions (PIs) are an important aspect of positive psychology, designed to teach people how to think, feel, and behave in a way that boosts positive feelings and positive behaviors toward others [7,8]. The goals of PIs should target well-being. Moreover, PIs must operate via mechanisms that are known to promote positive emotions, behaviors, and thoughts rather than fixing deficits or addressing maladaptive patterns [3]. Unlike conventional psychotherapy, which is aimed primarily at helping patients to cope with negative emotions, PIs have a wide range of applications and are not limited to therapeutic contexts. Individuals can practice on their own without a therapist, or they can seek out the help of various programs or life-coaches. At the same time, PIs do not require as much money and time as conventional medical treatments and carry no stigma or drug side effects [9]. A number of studies have already demonstrated that PIs can enhance individual well-being and relieve depression [2,8,10,11]. Indeed, the results of one meta-analysis revealed that PIs have a moderate effect on both subjective and psychological well-being and have a large effect on depression and anxiety [12].

Among the many evidenced-based positive interventions are those that focus on savoring. A number of previous studies have investigated the effects of savoring and used savoring exercises as part of many multi-component PIs [2,12]. Despite their promise, few studies have designed PIs that apply savoring-related strategies via social media. In this research we began with the idea that social media or social networking sites (SNSs) might provide an important means of delivering savoring interventions. For one, Bryant and Veroff [4] suggested that “sharing positive events with others” should be an important means by which people can savor life. Of course, SNSs are a prominent medium for such sharing, seemingly making them an ideal way to engage in the “sharing with others” strategy. Bryant and Veroff [4] also argued that savoring strategies involve “memory building.” The sharing of photos and updates on SNSs is likely an important avenue for such memory building. This is because SNSs facilitate responses from friends, leading to reminiscence and recall, chaining of experiences, and longer-term reverberations around the person’s social network, likely enhancing savoring even more. Many PIs require the participant to keep records of savoring interventions and engage in self-disclosure. This can be an inconvenience, which reduces compliance with researcher requests. However, SNSs have become the most common media for people to make a spontaneous record of their daily lives [13]. In view of the above, combining SNSs and an intervention to increase savoring should be a feasible PI approach.

## 2. Literature Review

### 2.1. Research Involving Savoring Intervention

The analytical results of our meta-analysis revealed that positive interventions (PIs) can be effective at enhancing well-being and reducing depressive symptoms in both Western and non-Western countries [12]. Indeed, a number of previous studies have shown that PIs that employ savoring have the potential to enhance positive affect (PA), reduce negative affect (NA), and improve depressive symptoms in various populations [2,4,14]. Relevant studies are briefly described over the following.

Seligman et al. conducted a savoring intervention for adults called “Three Good Things.” The results of this work indicated that savoring interventions promote positive emotions, reduce depression, and have long-term effects that can last up to six months [2]. Another investigation, performed by Bryant and Veroff [4], asked participants to take twenty-minute walks every day and look for positive events (such as seeing small animals, noticing the sunset, or hearing music). The results of this study showed that savoring interventions promote happiness.

Kurtz [15] derived an intervention activity for college students called “mindful photographs.” For this activity, college students were required to take meaningful photographs of specific subjects (e.g., their campus or friends) and, in so doing, concentrate on revealing creativity and beauty. Participants who took mindful photographs reported a significantly more positive mood compared to participants who took neutral, factual photographs. Among elderly adults, Geiger, Morey, and Segerstrom [16] found that if an individual is able to experience savoring, he or she will be more able to focus on happiness in the present, which also promotes health. Finally, Smith and Hanni [14] showed that savoring interventions improved depressive symptoms and happiness in elderly adults. Furthermore, in a 2012 study by Hurley and Kwon [17] that investigated “savoring-the-moment interventions,” participants in the intervention group experienced significant decreases in depressive symptoms and NA compared to participants in the control group; however, PA did not differ between groups.

Nonetheless, some studies have found that certain types of savoring interventions did not promote PA. For example, in research by Lyubomirsky et al. [10], different ways of savoring (i.e., writing, talking, and thinking) led to different effects. Specifically, results of that research indicated that thinking (i.e., simply being more mindful about the positive attributes of an experience) was effective at enhancing well-being; however, writing and talking did not have significant effects. Lyubomirsky et al. [10] also found that savoring interventions that involved writing did not improve PA. They argued that the organized and systematic nature of writing and talking may interfere and be somewhat incompatible with the maintenance of positive emotions. Conversely, Seligman et al. [2] found that participants who wrote about three positive events showed improved happiness, and the effects lasted six months.

As a result of inconsistent research findings, whether savoring interventions that employ writing can improve well-being is still unclear.

### 2.2. Social Network Sites and Savoring Intervention

Research has shown that internet-based PIs can increase positive emotions and reduce depression [2,18]. Among internet applications, SNS platforms could be promising tools for the effective implementation of interventions [13,19]. Using SNS has become routine in the lives of college students [20,21], and positive online interactions on SNS platforms have been found to increase social support and reduce depression. Furthermore, young people may be more willing to disclose information on an SNS than they are in person [19,20].

Social network interventions have been successfully used to positively impact a range of health-related behaviors, such as diabetes education, smoking, exercise, and dieting [19,22]. Social networking interventions based on cognitive behavioral therapy (CBT) have been tested in young people and have shown promising results in terms of reducing depression [23]. However, although many types of effective SNS interventions focus on improving health-related behaviors [19], fewer focus on improving psychological issues. Furthermore, most existing studies on social networking interventions have investigated interventions based on CBT rather than interventions based on savoring theory [23]. Yu [13] previously developed Facebook-based PIs, named “photo diary” and “expression of gratitude,” and found that they were effective in contributing to well-being. However, Yu’s interventions did not involve savoring theories; they were based on theories of “strengths and virtues” and “gratitude.” Indeed, few studies have investigated the effects of savoring interventions that employ an SNS. As Rice et al. [23] reported, more research is needed to elucidate the potential uses of social networking in interventions.

To address this research gap, we conducted a study that investigated SNS-based savoring interventions. As noted, previously reported effects of savoring interventions that employ writing are inconsistent. SNS platforms, such as Facebook, are popular, and Facebook walls provide a convenient way for college students to “write” (i.e., by making a post). When posts are “liked” or continuously responded to, prolonged positive emotions can result. However, whether savoring interventions can improve their effectiveness is in need of further investigation. In the current research, we built upon the “Three Good Things” methodology, modifying it to be appropriate for Facebook use. We believe that this modification should be effective because Facebook is extremely popular and widely used. Indeed, people already use Facebook to share and savor their lives, and users are accustomed to receiving positive responses (i.e., “likes”). Our study sought to capitalize on these facts.

## 3. Methods

### 3.1. Participants

The 122 initial participants of the study (41 men and 81 women) were student volunteers recruited from 14 classes being held in five different Taiwanese Universities (average age = 20.53, SD = 0.66). During the recruitment process, a research assistant explained the concept of savoring and the procedures, three-week duration, and data confidentiality of the experiment. Willing students were asked to sign a consent form and to supply an email address. The Institutional Review Board of National Chung Cheng University granted ethical approval to carry out the study (Ethical Application Ref: CCUREC-106021601). Informed consent was obtained from all participants. All methods were performed in accordance with the relevant guidelines and regulations of the institution.

Participants were emailed an initial survey and instructions, and also received a post-test survey three weeks later, immediately following completion of the PI. They were also emailed a final follow-up survey seven weeks later, four weeks after completion of the PI. To reduce attrition and motivate the participants, a gift certificate was promised at the end of the experiment, to the winner in a random draw. A total of 105 participants remained at the post-test and 98 participants at the final follow-up. Finally, 49 participants remained in the follow-up for experimental group, but four of them were excluded from analysis due to incomplete response. For the control group, 49 participants remained in the follow-up and were analyzed. The dropout rates of the experimental and the control group are 19.7% and 19.7%, respectively.

### 3.2. Procedure

**Figure 1:**
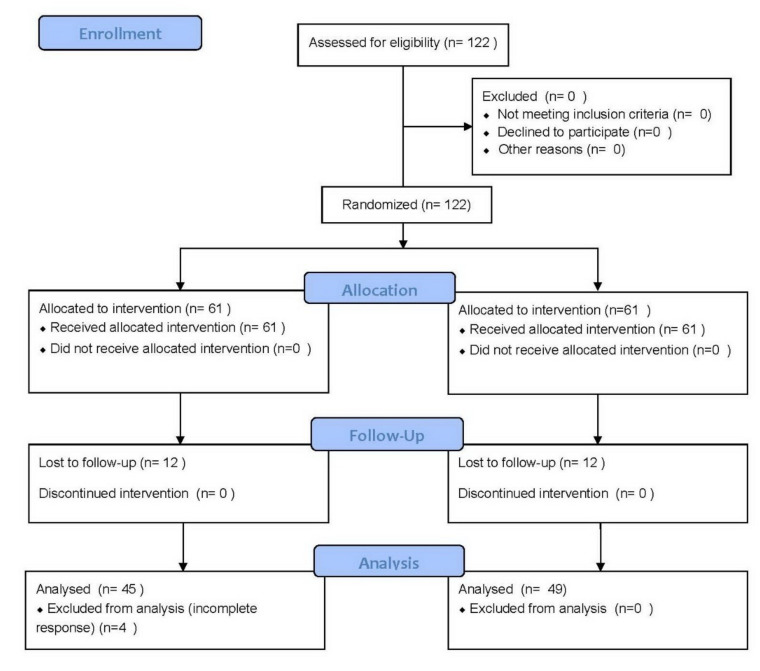
Trial flow diagram.

For allocation of the participants, a computer-generated list of random numbers was used. The initial participants being randomly assigned to either a savoring treatment group (*N* = 61) or to a no-treatment control group (*N* = 61). The CONSORT (Consolidated Standards of Reporting Trials) Flow Diagram is shown in Figure 1. The subjects were blinded to the treatment they received. This study met the basic requirements of the CONSORT 2010 checklist.

The savoring intervention was based on relevant research and strategies of savoring. During the intervention, experimental participants were asked to spend at least 20 min a day, at least three times a week, doing something that they like and enjoy. While doing the activity, they were asked to pay attention to the positive feelings that the activity brought them. After doing the activity, they were asked to describe and post these positive feelings on a social networking site, using text and/or photos. Then they recorded information about the experience using the “My Little Happy Things Record Form.” This information included the name of the activity, the time spend on the activity, the positive feelings and emotions derived from the activity, and the SNS used. Once completed, they returned the record form to the researcher via email. The experimental group was required to complete a PI for two weeks. Immediately following completion of the PI, participants took a post-test, and four weeks following completion of the PI, participants completed a follow-up test.

On the weekends during the experiment, the participants were asked to recall the little happy things that they had done that week and re-live the positive emotions that they had felt. They were also asked to complete and return a record about their savoring events and email it to the researcher. If the participant did not return a form on schedule, the researcher sent an email to remind them. Based on the responses given by the participants, Facebook was used 100% of the time, with a small portion of the participants (9.8%) using both Twitter and Facebook.

### 3.3. Measures

#### 3.3.1. Positive and Negative Affect Scales (PANAS)

We used the Chinese version of the positive and negative affect scales (PANAS), translated by Fang [24]. Fang also modified the original PANAS [25] from a five-point scale to a four-point scale to prevent central tendency errors. The results of a factor analysis by Fang indicated that the Chinese version of PANAS had good validity. Furthermore, Cronbach’s alpha values for the PA and NA scales were 0.86 and 0.87, respectively, indicating that the Chinese PANAS had good reliability as well [24]. In the current study, the PA showed alpha coefficients of 0.915 at the pre-test stage, 0.878 at the post-test stage, and 0.887 at the follow-up stage. The NA showed alpha coefficients of 0.847 at the pre-test stage, 0.860 at the post-test stage, and 0.881 at the follow-up stage, indicating good reliability.

#### 3.3.2. Center for Epidemiologic Studies Depression Scale (CES-D)

CES-D [26] is one of the most widely used depression scales in the world and has been translated and verified for reliability and validity in a number of languages. This study used the Chinese version of CES-D [27], which contains 20 question items scored on a four-point Likert scale. The analytical results revealed that CES-D Cronbach’s alpha equals 0.891, indicating good reliability. For this study, the CES-D had a coefficient alpha of 0.880 at the pre-test stage, 0.865 at post-test stage, and 0.892 at follow-up stage.

## 4. Results

### 4.1. Influence of the Intervention on Positive Affect

**Figure 2:**
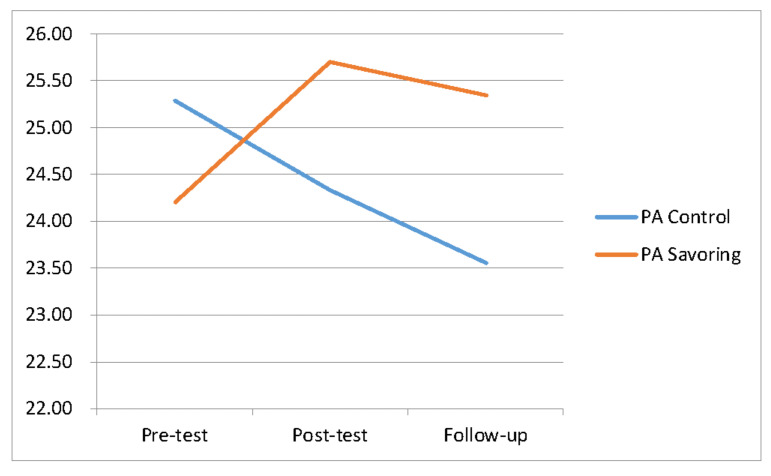
Changes in original mean score of PA during different stages.

The mean scores of the experimental group for PA in the pre-test, post-test, and follow-up tests were 24.20, 25.71, and 25.35, respectively, while the mean scores of the control group were 25.30, 24.33, and 23.55, respectively. The trends we observed are shown in Figure 2; and the means, and SDs for PA, NA, and CES-D are shown in Table 1.

An ANCOVA was performed to investigate group differences in PA over time. According to ANCOVA analysis, the covariate-adjusted mean of PA for the experimental group at the post-test stage was 26.15 (SD = 0.69), with a 95% CI of [24.79, 27.51], while for the control group, the covariate-adjusted mean of PA was 23.91 (SD = 0.67), with a 95% CI of [22.59, 25.24]. Significant differences were found (F (1, 102) = 5.38, *p* = 0.02 < 0.05, eta square = 0.05), suggesting that the mean PA scores for the experimental group were higher than those of the control group. Nonetheless, the value of eta square was between 0.01 and 0.06, indicating that the effect size was small at the post-test stage [28].

At the follow-up stage, the covariate-adjusted mean of PA for the experimental group was 25.84 (SD = 0.71), with a 95% CI of [24.45, 27.24], while for control group, the covariate-adjusted mean of PA was 23.05 (SD = 0.71), with a 95% CI of [21.65, 24.45]. Significant differences were also found at this stage (F (1, 95) = 7.69, *p* = 0.1 < 0.05, eta square = 0.075), which suggests that the effects of savoring intervention continued from the post-test stage to the follow-up stage. The eta square was 0.075, which is indicative of a medium effect size. This suggests that not only did the positive influence of the savoring intervention continue, it also grew in magnitude.

### 4.2. Influence of the Intervention on Negative Affect

**Figure 3:**
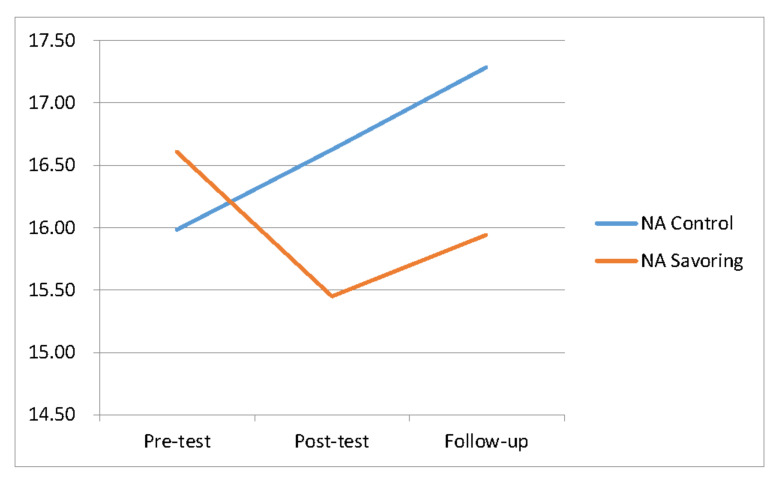
Changes in original mean score of NA during different stages.

Results from the NA scale showed that the mean scores of the experimental group in the pre-test, post-test, and follow-up tests were 16.61, 15.45, and 15.94, respectively, while the mean scores of the control group were 15.98, 16.63, and 17.29, respectively. The trends we observed are as shown in Figure 3 and descriptive statistics are shown in Table 1.

An ANCOVA was performed to investigate group differences on the NA over time. According to ANCOVA analysis, the experimental group’s covariate-adjusted mean of NA at post-test stage was 15.23 (SD = 0.60), with a 95% CI of [14.04, 16.43], while that of control group was 16.83 (SD = 0.59), with a 95% CI of [15.67, 18.00]. At the time of the post-test, no significant differences were found (F (1, 102) = 3.59, *p* = 0.06 > 0.05), although the *p*-value (0.06) was very close to 0.05.

At the follow-up stage, the experimental group’s covariate-adjusted mean of NA was 15.63 (SD = 0.76), with a 95% CI of [14.13, 17.14], while that of control group was 17.59 (SD = 0.76), with a 95% CI of [16.09, 19.10]. No significant differences were found (F (1, 95) = 3.26, *p* = 0.07 > 0.05). The results mean that the mean scores of NA of the experimental group did not differ from those of control group during both stages. The savoring intervention did not reduce the NA of the participants.

### 4.3. Influence of Intervention on Depression

**Figure 4:**
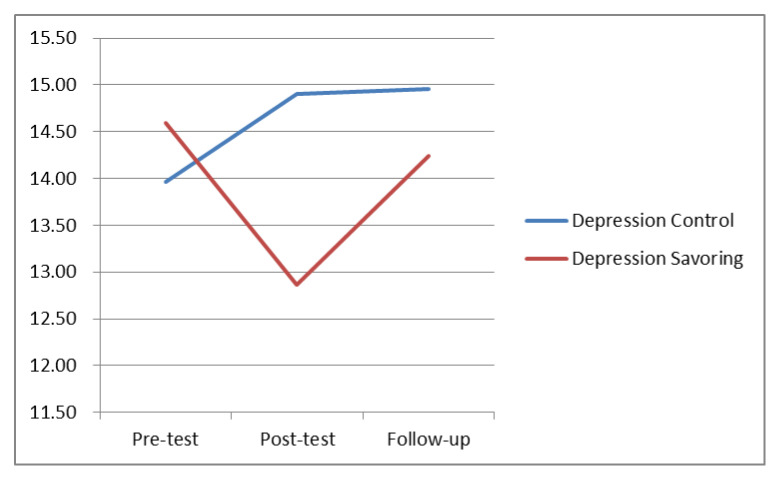
Changes in original mean score of CES-D during different stages.

Results from the CES-D scale showed that the mean scores of the experimental group in the pre-test, post-test, and follow-up tests were 14.59, 12.86, and 14.24, respectively, while the mean scores of the control group were 13.97, 14.89, and 14.96. The trends we observed are as shown in Figure 4 and descriptive statistics are shown in Table 1.

An ANCOVA was performed to investigate group differences on the CES-D over time. In the post-test stage, experimental group’ covariate-adjusted mean of CES-D was 12.42 (SD = 0.95), with a 95% CI of [10.54, 14.30], while that of control group was 15.33 (SD = 0.92), with a 95% CI of [13.49, 17.16]. Significant differences were found (F (1, 102) = 4.770, *p* = 0.031 < 0.05, eta square = 0.045) with a small effect size.

At the follow-up stage, experimental group’ covariate-adjusted mean of CES-D was 13.73 (SD = 1.17), with a 95% CI of [11.42, 16.04], while that of control group was 15.48 (SD = 1.17), with a 95% CI of [13.16, 17.79]. No significant differences were found at the follow-up stage (F (1, 95) = 1.109, *p* = 0.295 > 0.05). The results indicate that the positive savoring intervention had an immediate effect for reducing depression, but the effects did not last till follow-up stage.

## 5. Discussion

We tested the idea that people can use social networking sites to savor positive experiences, thereby enhancing the effective duration of those experiences. It is very possible that SNS platforms can improve the implementation of savoring interventions. Bryant and Veroff [4] proposed ten strategies to enhance savoring: sharing with others, memory building, self-congratulation, sensory-perceptual sharpening, comparing, absorption, behavioral expression, temporal awareness, counting blessings, and avoiding kill-joy thinking. Bryan and Veroff [4] also proposed four specific methods to prolong savoring: reminiscence and recall, chaining, sharing after the moment, and celebration. Seen in this light, it is likely that our intervention participants were simultaneously engaged in many of these strategies or methods. While implementing the general savoring strategy of “sharing with others,” they were “absorbed” in typing out Facebook posts, which promoted “reminiscence and recall,” “memory building,” “sharing after the moment,” and “counting blessings.” For these reasons, it is not surprising that the intervention boosted PA, validating SNS as an effective way to savor life-experiences and to prolong positive emotion, similar to the findings of [4,10,29].

The results of this study indicated that the savoring intervention that we applied can enhance PA, which is similar to findings reported by Bryant and Veroff [4], Smith et al. [29], and Hendriks et al. [12]. Moreover, our results confirmed that savoring interventions that employ writing promote PA. However, our results contradict findings by Lyubomirsky et al. [10], in which savoring interventions that employed writing did not promote PA. It is possible that writing about positive events encourages the construction of a logical narrative, which enhances the maintenance of positive emotions. Moreover, writing activities that involve Facebook posts are easy and interesting for college students [21]. We thus posit that SNS facilitates savoring interventions that employ writing by making writing activities more simple and interesting.

We found that although SNS-based savoring interventions promoted PA, it did not simultaneously reduce NA. It is possible that people often count their blessings instead of unloading their problems on Facebook. Facebook users mostly use status updates to share positive emotions [30], and users are often more likely to express positive emotions than negative emotions because they wish to present an image of good emotional well-being on Facebook [31]. One study found that participants who shared and savored positive events on Facebook enjoyed an increase in PA. In the current work, the effect sizes of PA were found to increase from the post-test stage to the follow-up stage. We suspect that “likes” and other responses to Facebook posts prolonged the positive emotions experienced by participants. Conversely, savoring interventions were less effective at improving NAs.

Our study also found that the internet-based positive savoring intervention could somewhat relieve depression, which is similar to the result obtained by Seligman et al. [2] and Kahrilas et al. [32]. What is different is that Seligman et al. employed group positive psychotherapy as well as six types of interventions, and their savoring intervention was only one of six that obtained significant results. In contrast, our study discovered that a single savoring intervention alone could be effective. Moreover, this intervention method allows individual freedom without the restrictions of group consultation.

## 6. Limitations

This study employed an inactive (do-nothing) control group, which may have had a confounding influence on our results. Future studies could further this research by employing control groups that receive a “neutral” intervention (i.e., an intervention in which participants do a non-savoring exercise), thereby ensuring that both control and experimental groups undergo more equivalent treatments.

This study has other limitations, including that it employed a single sample of Taiwanese college students, it relied on self-report measures, and a single intervention was employed. Further studies could attempt to sample different cultural groups, populations, age cohorts, or personalities, to establish generalizability. They could also compare Facebook savoring with other positive interventions, such as writing gratitude letters or extending forgiveness, to evaluate their relative effectiveness. Additionally, with regard to participant gender, there were more women than men in this study. It may be because the intervention is more introspective and psychology oriented. The unbalanced gender distribution may influence the generalizability.

Future studies can also extend the duration of interventions. The intervention in this study lasted three weeks, and significantly decreased depression and increased PA were found in the post-test stage. However, the depression-relieving effects did not last to the follow-up stage. Other savoring interventions only continued for three days, with 15 min of activity per day [6], or for a week, with daily tasks [2,4], or for two weeks, with tasks twice a week [15]. Research is still ongoing to find the appropriate frequency and duration for interventions. The appropriate frequency might maintain immediate and lasting effects but also prevent weariness and habituation. Moreover, further studies should consider other SNSs, such as Instagram, which is popular among college students.

## 7. Conclusions

This study designed a Facebook-based savoring intervention and exercised on university students in Taiwan. The results showed Facebook-based savoring intervention enhanced positive affect in both a post-test and a final follow-up, and also reduced depression in the post-test. The results showed SNS platforms can improve the implementation of savoring interventions.

## Figures and Tables

**Table 1:** Summary of Means and Standard Deviations for scores on positive affect (PA), negative affect (NA), and Center for Epidemiologic Studies Depression Scale (CES-D).

		Pre-Test	Post-Test	Follow-Up
Measure	Condition	M (SD)	M (SD)	M (SD)
PA	Exp	24.20 (6.07)	25.71 (5.44)	25.35 (4.59)
	Con	25.30 (6.30)	24.33 (5.84)	23.55 (6.28)
NA	Exp	16.61 (5.17)	15.45 (4.73)	15.94 (4.92)
	Con	15.98 (4.61)	16.63 (4.76)	17.29 (5.86)
CES-D	Exp	14.59 (8.58)	12.86 (8.53)	14.24 (8.43)
	Con	13.97 (8.24)	14.91 (7.46)	14.96 (8.61)

Note: PA = positive affect scale; NA = negative affect scale; CES-D = Center for Epidemiologic Studies Depression Scale; Exp = experimental group; Con = control group.

## References

[1] Seligman M., Csikszentmihalyi M. (2000). Positive psychology: An introduction. Am. Psychol..

[2] Seligman M.E.P., Steen T.A., Park N., Peterson C. (2005). Positive Psychology Progress: Empirical Validation of Interventions. Am. Psychol..

[3] Schueller S.M., Parks A.C. (2014). The science of self-help. Eur. Psychol..

[4] Bryant F.B., Veroff J. (2007). Savoring: A New Model of Positive Experience.

[5] Bryant F.B. (2003). Savoring Belief Inventory(SBI): A scale for measuring beliefs about savoring. Int. J. Ment. Health Nurs..

[6] Lyubomirsky S., Sousa L., Dickerhoof R. (2006). The costs and benefits of writing, talking, and thinking about life’s triumphs and defeats. Pers. Soc. Psychol..

[7] Duckworth A., Steen T., Seligman M. (2005). Positive Psychology in Clinical Practice. Annu. Rev. Clin. Psychol..

[8] Sheldon K.M., Lyubomirsky S. (2006). Achieving Sustainable Gains in Happiness: Change Your Actions, Not Your Circumstances. J. Happiness Stud..

[9] Layous K., Chancellor J., Lyubomirsky S., Wang L., Doraiswamy P.M. (2011). Delivering happiness: Translating positive psychology intervention research for treating major and minor depressive disorders. J. Altern. Complem. Med..

[10] Lyubomirsky S., Sheldon K.M., Schkade D. (2005). Pursuing happiness: The architecture of sustainable change. Rev. Gen. Psychol..

[11] Sin N.L., Lyubomirsky S. (2009). Enhancing well-being and alleviating depressive symptoms with positive psychology interventions: A practice-friendly meta-analysis. J. Clin. Psychol..

[12] Hendriks T., Schotanus-Dijkstra M., Hassankhan A., Graafsma T., Bohlmeijer E., de Jong J. (2018). The efficacy of positive psychology interventions from non-Western countries: A systematic review and meta-analysis. Int. J. Qual. Stud. Health Well-Being.

[13] Yu S.C. (2020). Does using social network sites reduce depression? An example of Facebook-based positive interventions. Int. J. Technol. Hum..

[14] Smith J.L., Hanni A.A. (2009). Effects of a savoring intervention on resilience and well-being of older adults. J. Appl. Gerontol..

[15] Kurtz J.L. (2012). Seeing through new eyes: An experimental investigation of the benefits of photography. J. Basic Appl. Sci..

[16] Geiger P.J., Morey J.N., Segerstrom S.C. (2017). Beliefs about savoring in older adulthood: Aging and perceived health affect temporal components of perceived savoring ability. Pers. Individ. Differ..

[17] Hurley D.B., Kwon P. (2012). Results of a study to increase savoring the moment: Differential impact on positive and negative outcomes. J. Happiness Stud..

[18] Corno G., Etchemendy E., Espinoza M., Herrero R., Molinari G., Carrillo A., Baños R.M. (2018). Effect of a web-based positive psychology intervention on prenatal well-being: A case series study. Women Birth.

[19] Latkin C.A., Knowlton A.R. (2015). Social network assessments and interventions for health behavior change: A critical review. J. Behav. Med..

[20] Ellison N.B., Steinfield C., Lampe C. (2007). The benefits of Facebook “friends:” Social capital and college students’ use of online social network sites. JCMC.

[21] Yu S.C. (2015). Happiness or addiction: An example of Taiwanese college students’ use of Facebook. Int. J. Technol. Hum..

[22] Gabarron E., Årsand E., Wynn R. (2018). Social media use in interventions for diabetes: Rapid evidence-based review. J. Med. Internet. Res..

[23] Rice S.M., Goodall J., Hetrick S.E., Parker A.G., Gilbertson T., Amminger G.P., Alvarez-Jimenez M. (2014). Online and social networking interventions for the treatment of depression in young people: A systematic review. J. Med. Internet Res..

[24] Fang T.W. (2012). The Relations among Perfectionism, Learning Problem, and Positive and Negative Affect: The Mediating Effect of Rumination. J. Educ. Psychol..

[25] Watson D., Clark L.A., Tellegen A. (1988). Development and validation of brief measures of positive and negative affect: The PANAS scales. J. Pers. Soc. Psychol..

[26] Radloff L.S. (1977). The CES-D scale: A self-report depression scale for research in the general population. Appl. Psychol. Meas..

[27] Yu S.C., Yu M.M. (2007). Comparison of Internet-Based and Paper-Based Questionnaires in Taiwan Using Multi-Sample Invariance Approach. Cyberpsychol. Behav. Soc. Netw..

[28] Cohen J. (1988). Statistical Power Analysis for the Behavioral Sciences.

[29] Smith J.L., Harrison P.R., Kurtz J.L., Bryant F.B., Parks A.C., Schueller S.M. (2014). Nurturing the capacity to savor: Interventions to enhance the enjoyment of positive experiences. The Wiley Blackwell Handbook of Positive Psychological Interventions.

[30] Vermeulen A., Vandebosch H., Heirman W. (2018). Smiling, venting, or both? Adolescents’ social sharing of emotions on social media. Comput. Hum. Behav..

[31] Qiu L., Lin H., Leung A.K., Tov W. (2012). Putting their best foot forward: Emotional disclosure on Facebook. Cyberpsychol. Behav. Soc. Netw..

[32] Kahrilas I.J., Smith J.L., Silton R.L., Bryant F.B. (2020). Savoring the moment: A link between affectivity and depression. Int. J. Qual. Stud. Health.

